# Spectral Multiplexing of Fluorescent Endoscopy for Simultaneous Imaging with Multiple Fluorophores and Multiple Fields of View

**DOI:** 10.3390/bios13010033

**Published:** 2022-12-27

**Authors:** Bjorn Paulson, Saeed Bohlooli Darian, Youngkyu Kim, Jeongmin Oh, Marjan Ghasemi, Kwanhee Lee, Jun Ki Kim

**Affiliations:** 1 Biomedical Engineering Research Center, Asan Institute for Life Science, Asan Medical Center, Seoul 05505, Republic of Korea; 2 Department of Convergence Medicine, University of Ulsan College of Medicine, Seoul 05505, Republic of Korea; 3 Department of Physics, Yonsei University, Seoul 03722, Republic of Korea

**Keywords:** microendoscopy, multichannel, attachable endoscope system, spectral multiplexing, dual endoscope, lens relay, intravital imaging, dual-site imaging

## Abstract

Complex clinical procedures and small-animal research procedures can benefit from dual-site imaging provided by multiple endoscopic devices. Here, an endoscopic system is proposed which enables multiple fluorescence microendoscopes to be spectrally multiplexed on a single microscope base, enabling light sources and optical relays to be shared between endoscopes. The presented system is characterized for resolution using USAF-1951 resolution test charts and for modulation transfer function using the slanted edge method. Imaging is demonstrated both directly and with microendoscopes attached. Imaging of phantoms was demonstrated by targeting USAF charts and fiber tissues dyed for FITC and Texas Red fluorescence. Afterwards, simultaneous liver and kidney imaging was demonstrated in mice expressing mitochondrial Dendra2 and injected with Texas Red-dextran. The results indicate that the system achieves high channel isolation and submicron and subcellular resolution, with resolution limited by the endoscopic probe and by physiological movement during endoscopic imaging. Multi-channel microendoscopy provides a potentially low-cost means of simultaneous multiple endoscopic imaging during biological experiments, resulting in reduced animal harm and potentially increasing insight into temporal connections between connected biological systems.

## 1. Introduction

Dual endoscopy is a clinical technique in which two endoscopes are used simultaneously to resolve a surgical challenge. Dual endoscopy is most commonly reported in the management of challenging airways [1,2], where an endoscope and a laryngoscope are used in tandem: one scope provides the surgeon with guidance information, a stabilizing mount point, or illumination for the other. Complex procedures based on simultaneous use of multiple scopes have also been related in the treatment of GI obstructions [3], in the reduction of gastric volvulus [4], and in biliary drainage [5]. In percutaneous kidney stone nephrolithotomy, the use of multiple scopes has been identified as predictive of successful kidney stone freeing [6].

Preclinical and biological studies of small animals also benefit from dual endoscopy. In neuroscience, a continuing challenge is the correlation of signals across areas of the mouse brain, as standard probes and microscope objectives have too large a mechanical footprint to be used in tandem to image nearby areas of the cortex [7], and thus the potential for dual-site microendoscopy motivates continued miniaturization [8]. However, these studies have been limited to rigid gradient-index probes embedded in the mouse cortex. While even without dual endoscopy, microendoscopes enable minimally invasive and longitudinal observations which reduce the number of study animals needed [9,10,11], the development of a more adaptable multi-fluorescence endoscope system would enable the study of any potentially interacting multi-organ acute pathologies throughout the body of the small animal.

In this study, an endoscopic system is proposed which enables simultaneous visualization from multiple locations via spectral multiplexing. The system is composed of attachable modules which can be assembled around a microscope base (already available in most intravital imaging laboratories), and requires a single broadband illumination source. We demonstrate submicron-scale resolutions of 0.94 and 0.98 µm without endoscopic probes and micron-scale resolutions with both rigid gradient-index and flexible fiber bundle microendoscopic probes. We find that crosstalk and fluorescence imaging are both minimal following appropriate filter selection. The endoscopic system is demonstrated in a GFP-fluorescent and Texas Red-fluorescent mouse model, revealing subcellular features, microcapillary structure, and small organoids. The system performance is further demonstrated on test targets and fluorescently dyed microfibers. The spectrally multiplexed endoscopy system is well suited for investigating the temporal interactions between disparate organ systems or between different sites on the same organ during *in vivo* small animal studies, and is promising for applications in preclinical studies of both biological and medical interest.

## 2. Materials and Methods

### 2.1. Illumination and Excitation Filtering

**Figure 1:**
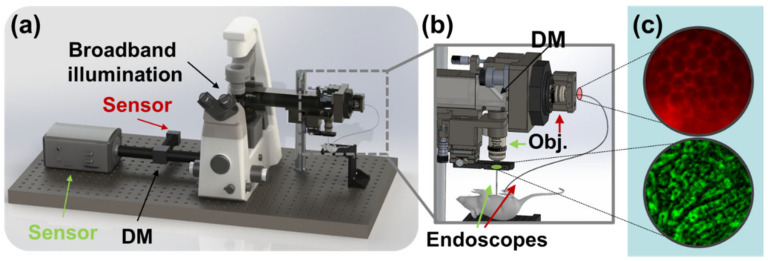
Rendering and overview of the experiment. (**a**) Broadband illumination is multiplexed between two channels and separated prior to sensing by dichroic mirrors (DM). (**b**) Dichroic mirror at distal end of lens relay assembly enables microendoscopic probes to be illuminated simultaneously and used to image different fields of view on the target animal. (**c**) Images of biological interest are captured in red (top) and green (bottom) fluorescence channels.

**Figure 2:**
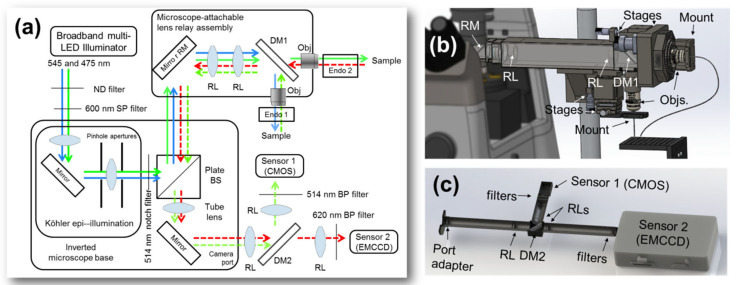
Experimental setup details. (**a**) Schematic of the experiment. (**b**) Illumination is multiplexed between GFP (<532 nm) and Texas Red-dextran (>532 nm) channels by a dichroic mirror (DM1) distal to the illumination lens relay, from which individual microendoscopic probes are mounted. Returning signals are multiplexed for transport through the microscope base. (**c**) The sensor assembly consists of another lens relay containing a dichroic mirror which separates the signals (DM2) for detection as well as several filters to isolate the channels. BP, bandpass filter; BS, beam splitter; DM, dichroic mirror; ND, neutral density filter; Obj., objective lens; RL, relay lens; RM, proximal relay mirror; SP, short-pass filter.

An optical system comprising multiple micro-endoscopes and multiple imaging cameras was assembled around an inverted microscope base, as shown as a rendering in Figure 1 and as shown in schematic in Figure 2. A broadband LED illuminator with seven LEDs from 450 nm to 750 nm (Xylis X-Cite, Excelitas, Waltham, MA, USA) was coupled to the Köhler epi-illumination channel of the inverted microscope (TE-2000U, Nikon, Japan). Input light was filtered in the Köhler assembly to eliminate light which would overlap the emission wavelengths via two filters: a 514 nm notch filter (17 nm FWHM bandwidth, Thorlabs, Newton, NJ, USA) and a 600 nm shortpass filter (FELH600, Thorlabs). A 50:50 non-polarizing fused silica plate beam splitter (Thorlabs) was used to redirect the illumination beam through the microscope turret into a lens relay assembly.

### 2.2. Attachable Lens Relay Assembly and Spectral Multiplexing

The lens relay assembly was fixed relative to the microscope base and was used to relay light between the microscope and the endoscopes for both illumination and imaging. The relay system, its principle, and a method for the construction of similar relay assemblies have been related in previous publications [12,13,14]. Briefly, a proximal mirror redirects the collimated beam from the microscope turret into a horizontal 4*f* lens relay, and a second distal mirror redirects the beam into the objective lens. Where previous lens relay assemblies have allowed rotation of the distal mirror for horizontal imaging, the device used in this study provided that the second mirror could be removed or replaced with a semitransparent optic (Figure 1b, Figure 2b).

Thus uniquely when compared to previous lens relay systems, the present lens relay assembly also provides the ability to multiplex (split) the light into each imaging channel, via a distal dichroic mirror (532 nm cutoff, Chroma, Bellows Falls, VT, USA) placed at the distal end of the lens relay assembly. With both horizontal and vertical channels, the relay system enables convenient *in vivo* imaging of lab animals. In horizontal mode, the beam is well-suited for coupling into microendoscopes for esophageal and colorectal imaging, while the vertical beamline is well-suited for microendoscopic intercranial, renal, and hepatic imaging in mice. In our application, the “green” channel (*λ* < 532 nm for imaging of FITC and GFP) was directed down the vertical path for imaging the exteriorized liver, and the “red” channel (*λ* > 532 nm for imaging of TRITC and Texas Red) was directed along the horizontal for imaging the exteriorized kidney via a flexible endoscope probe.

### 2.3. Objectives and Endoscopes

Two microendoscopes were used to demonstrate the endoscopic system: a 5 cm gradient index (GRIN) endoscope, consisting of a 1-pitch GRIN relay lens flanked by two quarter-pitch GRIN imaging lenses (SELFOC, Nippon Sheet Glass Co., Ltd., Kanagawa, Japan), and a flexible fiber bundle endoscope, consisting of a bundle of 30,000 optical fibers (Fujikura image fiber, via Myriad fiber, Dudley, MA, USA), and again capped on each end by GRIN imaging lenses. Fibers within the bundle are arranged in a roughly hexagonal pattern with a pitch of 3.3 µm, for total bundle diameter 650 µm and length 1 m. Microendoscope probes were mounted in threaded grooves to assist in alignment relative to the beam paths.

Light was coupled into and out of endoscopes by two objective lenses (Obj.), which were mounted on threaded adapters along each channel (Figure 1b): 40× Objs. for coupling into GRIN lenses (0.6 NA, Olympus, Japan) and a 20× Obj. (0.4 NA, Olympus) for coupling into flexible fiber bundles, where a wider field of view was preferred, as resolution is limited by fiber pitch. Measurements without endoscopes were performed with 40× objectives, except where otherwise specified.

### 2.4. Return Imaging Path

Following illumination of samples either directly through the objectives or through attached endoscopes, fluorescence signals were collected from the samples. Light collected by the objectives was guided into the dichroic beam splitter in the distal mirror position of the lens relay assembly, where it was multiplexed before reverse passage through the relay. Inside the microscope body, light reflects off the 50:50 plate beam splitter and is redirected toward the eyepiece and the camera port. Another lens relay assembly containing a dichroic beam splitter was attached to the camera port, to extend the image plane from the standard C-mount distance to a distance of roughly 380 mm from the microscope body, providing space for the demuxing dichroic mirror. As shown in detail in Figure 2c, this relay consisted of three convex lenses with antireflective coatings for the visible region (*f* = 150 mm, *f* = 125 mm, Thorlabs), a dichroic mirror paired to that in the endoscope relay (cutoff wavelength 532 nm, Chroma), and a 750 nm shortpass filter (Asahi Spectra, Tokyo, Japan), mounted in lens tubes (Thorlabs). The dichroic mirror in the camera relay assembly demuxes the signal, sending each spectral component of the beam to a different camera to enable simultaneous imaging.

### 2.5. Imaging Sensors

Two different cameras were used to demonstrate the principle of the device. On the “green” channel, set up for detection of FITC and GFP/Dendra2, a 1616 × 1240 CMOS camera (Teledyne FLIR, Thousand Oaks, CA, USA) was installed, following filtering by a 514 nm bandpass (BP) filter. On the “red” channel, set up for detection of TRITC and Texas Red, an electronically multiplying charged-coupled device (EMCCD) was installed (iXon Life, Andor, Belfast, UK) which captured images at 1024 × 1024 pixels following filtering for the emission band of Texas Red (620 nm BP, Thorlabs).

### 2.6. Imaging Phantom Samples, Reflective and Fluorescent

**Figure 3:**
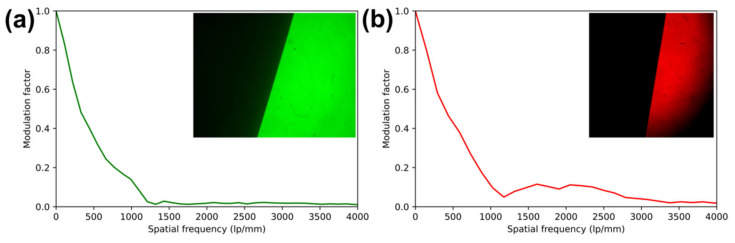
Modulation transfer functions (MTF) of each channel, as captured simultaneously and measured by the slanted edge method using reflective USAF targets. Images were captured in reflection with excitation filters removed. (**a**) green channel slanted-edge MTF analysis and (inset) contrast-normalized image of target. (**b**) red channel slanted-edge MTF analysis and (inset) contrast-normalized image of target.

**Figure 4:**
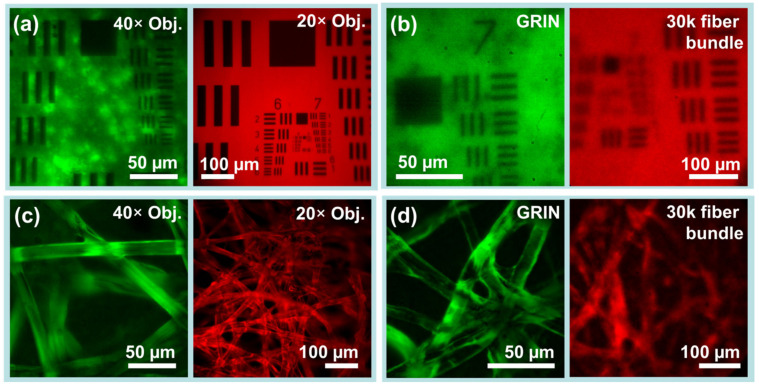
Phantom imaging demonstrates resolution and capabilities of the imaging system. (**a**,**b**) USAF-1951 resolution test charts establish practical fields of view and imaging resolution for each endoscope channel. (**a**) Low inter-channel cross-talk as revealed by USAF-1951 targets imaged through objective lenses. (left) Fluorescent-backed positive fluorescent test chart in green beam path. (right) Paper-backed positive fluorescent test chart in red beam path. (**b**) The same test charts simultaneously imaged through (left) a 5 cm GRIN microendoscope probe of 1 mm diameter and (right) through a 30k flexible fiber bundle endoscope probe of 0.65 mm diameter. (**c**,**d**) Fluorescently dyed lens paper which provides 3D structure similar to biological tissues. (**c**) Dyed lens papers imaged with objective lenses but without endoscopes. (**d**) The same papers imaged using GRIN and fiber bundle endoscope probes. Images captured in greyscale have been normalized and falsely colored for display.

The system was tested and profiled with samples in fluorescent and reflection modes. For the GFP/FITC channel (<512 nm), a USAF-1951 target with an FITC-dyed fluorescent backing was used to profile the channel and to verify fluorescence detection (#57855, Edmund Optics, Barrington, NJ, USA). As red or yellow-region targets were not available for the red channel, a non-fluorescent USAF-1951 target was used in the red channel (Figure 3a) with fluorescence by autofluorescence off a piece of paper placed behind the USAF-1951 target (Figure 3a). Channels were also profiled in reflectance mode, for which the red and green channels were profiled simultaneously following removal of the excitation filters from the Köhler epi-channel. (Insets, Figure 4).

### 2.7. Animal Experiments

A doubly fluorescent mouse model was prepared for demonstration of simultaneous multiple endoscopy. Male homozygous PhAM^excised^ mice expressing a mitochondrial-specific version of Dendra2 (JAX strain #018397, Jackson Laboratory, Bar Harbor, ME, USA) were anesthetized with an intraperitoneal solution of 60 μL zolazepam-tiletamine (Zoletil 50, Virback, Carros cedex, France) and 40 μL xylazine (Rompun, Elanco, Seoul, Republic of Korea) in 900 μL PBS. Protocols called for 200 μL of the solution to be used to anesthetize each mouse, assuming an average weight of 20 g. Following anesthetization, mice were shaved, liver and kidney were exteriorized, and roughly five minutes prior to imaging, Texas Red-dextran (Thermofisher Korea, Seoul, Republic of Korea) was injected intravenously into the eye vein as one 300 µL dose of 10 mg/mL in phosphate-buffered saline. Mice were then placed on a heated pad under the lens relay, and the exteriorized organs were immobilized relative to the endoscope probe tips. Where immobilization was insufficient, manual gating was used: specific frames of low motion were selected from video. Mice were imaged within 30 min of injection and sacrificed after imaging. All animal experiments were approved by the Institutional Animal Care and Use Committee of the Asan Institute for Life Sciences, Asan Medical Center (2021-12-030), under the laws of the Republic of Korea.

### 2.8. Data Analysis

Images were captured from the sensors via USB interfaces, using Micromanager [15,16] on ImageJ [17]. For display, images were normalized to 8-bit depth excluding 0.3% of extreme pixel intensities, pseudocolor was added using lookup tables to distinguish images captured on the “red” and “green” channels, and images were cropped to a square frame. For phantom and *in vivo* images, background fluorescence and variable illumination was removed using a sliding paraboloid background removal algorithm [18,19], after which the image was normalized. For *in vivo* images, Contrast Limited Adaptive Histogram Equalization (CLAHE) was performed to enhance local feature contrast [20], and for images captured via fiber bundles, a 2-pixel Gaussian blur was applied to the whole image after all other transformations to attenuate the effects of pixel subsampling.

Modulation transfer functions were calculated using the slanted-edge method via the SE_MTF plugin [21,22] for ImageJ. The large squares from the reflective front surface of the USAF-1951 target were used as slanted edges.

## 3. Results

### 3.1. Multiple Microendoscope Probes on a Single Microscope Base

A microscope-based assembly was designed which supports multiple-view fluorescent endoscopy using a single illumination system. As the intended use of the system is to find biological connections between acute pathologies in small animal models, simultaneous dual use of microendoscopes is supported with each channel providing grooves for microendoscope alignment. As shown in Figure 2, the system provides for fine endoscope alignment in two ways: while both channels provide for a three-axis stage to focus objective lenses relative to the microendoscope face, the horizontal channel is placed on a rotary mount and provides an additional two lateral axes for wobble correction (centering the microendoscope in the rotary mount).

### 3.2. Crosstalk-Free Spectral Multiplexing

Simultaneous dual-channel operation is shown to be free of inter-channel crosstalk via USAF-1951 resolution test charts. As USAF-1951 targets in reflectance imaging provide much higher contrast than fluorescent biological samples, they provide a worst-case test of crosstalk. Even with excitation filters removed for reflectance imaging, the targets in Figure 3 showed no noticeable cross-talk. With excitation filters in place for fluorescence imaging, as shown in Figure 4a,b, again no cross-talk was observable.

### 3.3. System Resolution

The resolution of the imaging system was measured via the slanted-edge modulation transfer function (MTF) method [21] in reflectance and via direct observation of USAF-1951 test charts in fluorescence. For slanted-edge measurements, two USAF-1951 targets, one in each channel, were placed under 40× objectives and focused upon with excitation filters removed from the imaging system. The resulting best-focus images are shown as insets to the resulting slanted-edge MTF in Figure 3. These images were analyzed using the SE_MTF plugin for ImageJ [21]. Despite different imaging sensors used in each channel, MTF was found to be similar, with the MTF dropping below 0.5 at 317 line pairs per mm (lp/mm) in the green channel and 394 lp/mm in the red channel, below 0.2 at 777 and 840 lp/mm, and below 0.1 at 1070 and 1020 lp/mm in the green and red channels, respectively, corresponding to 0.94 and 0.98 µm resolutions.

Images of USAF-1951 test charts and fluorescently dyed lens paper as captured via each channel in each imaging condition are shown in Figure 4. When imaging without microendoscopes, the green beam path attains more than 228 lp/mm and the red beam path at least 645 lp/mm (with measurement limited by targets). Resolution through the flexible bundle microendoscope probe is limited to 64 lp/mm, but simultaneous rigid GRIN microendoscope probe resolution is greater than 161.3 lp/mm.

### 3.4. System Fields of View and Aberrations

The practical field of view (FOV) of the system in each channel is limited by the objectives used as well as by the endoscopes used. Practical fields of view are shown in Figure 4, and summarized quantitatively as follows. As measured along the edge of the square frame, the 40× objective on the green channel achieved a FOV of 186 µm, while the 20× objective on the red channel achieved a FOV of 643 µm. For GRIN lenses and fiber bundles, fields of view measured 133 µm and 364 µm, respectively.

Across the full field of view of the objectives on the USAF-1951 targets, a slight lateral tilt aberration was observed and can be attributed to use of the dichroic mirror. The image tilt was approximately 1° relative to when an aluminum mirror was used. (Not shown) Neither barrel nor pincushion aberrations were observed.

### 3.5. Imaging Phantoms through GRIN and Flexible Fiber Bundle Microendoscopes

Images of fluorescently dyed tissue paper captured via both channels are also shown in Figure 4. Fluorescent tissue paper is a good phantom for biological tissues, as unlike resolution test charts it has axial depth, and is thus subject to factors which complicate biological imaging such as out-of-focus elements and background fluorescence. Lens papers dyed with FITC and with Texas Red were imaged with objectives and with microendoscope probes. While images captured via objectives had much higher resolution and much finer features, in all cases, the individual fibers of the paper (~10 µm diameter) were clearly visible. Interestingly, imaging in the FITC channel via GRIN microendoscope probes attained better rejection of out-of-focus elements than via a 40× microscope objective.

### 3.6. Demonstration of Simultaneous Liver and Kidney Imaging in Mouse Model

**Figure 5:**
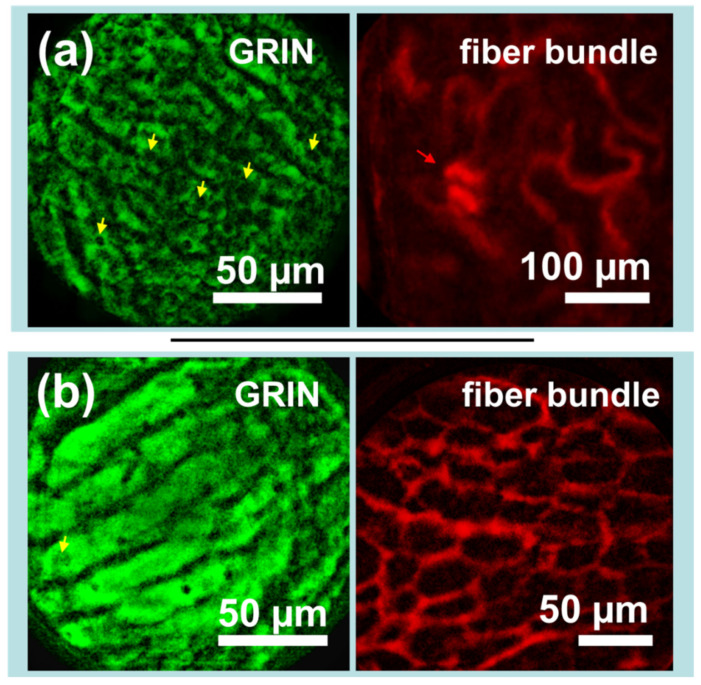
Sets of simultaneously captured *in vivo* images demonstrate the potential of the device for imaging the biological interactions between disparate organs. (**a**) The liver of a Dendra2 knock-in mouse (left) imaged along with the kidney vasculature of the same mouse after intravenous injection of Texas Red-dextran (right). (**b**) The liver of a Dendra2 knock-in mouse (left) imaged along with kidney adipose tissue vasculature after intravenous injection of Texas Red-dextran (right). Dendra2 images were captured by gradient index (GRIN) lens, while Texas Red images were captured via a flexible fiber bundle. Yellow arrows indicate potential lipid vacuoles or cellular nuclei, while the red arrow indicates likely glomerular structures. Images have been processed to remove background fluorescence, normalized, and falsely colored for display.

Images from the kidneys and livers of mice expressing mito-GFP were captured following injection with Texas Red-dextran. As seen in Figure 5a, images taken of the liver via GRIN lens microendoscope probes clearly show capillaries and cellular-level details, including many dark elliptical patches which are likely either lipid vacuoles or cellular nuclei (yellow arrows), while simultaneously captured images of the kidney show the looping vasculature characteristic of the kidney capillary network and what appear to be glomerular structures (red arrow). Figure 5b shows different perspectives of the same organ: the liver via GRIN lens and the vascularity of the kidney adipose tissue via fiber bundle. As the GFP image in Figure 5b was slightly overexposed, it is challenging to distinguish subcellular structures. However, the effect highlights the liver capillaries. The paired image shows the potential for measurement of liver adipocytes, which may have applications in the preclinical study of fatty liver diseases.

## 4. Discussion

The sensing system described in this study enables simultaneous fluorescent imaging at multiple locations *in vivo*, offering the potential to observe connections between intravital images of disparate organs, or to allow multiple simultaneous views of the same organ. Micron-scale resolutions are attained in each channel, with subcellular imaging demonstrated in a GFP channel and organoid imaging demonstrated in Texas Red. The device is suited for the longitudinal study of chronic disease, such as the connection between fatty liver disease and kidney function, or for the study of interactions between systems in models of acute pathologies, such as liver function during acute kidney failure.

Although the literature has a paucity of similar experiments which attempt dual-microendoscopy of different organs in small animals, dual-view fluorescence imaging of the same organ has been found useful, especially in small-animal neurology. Lecoq *et al.* [7] found dual-axis Ca^2+^ microendoscopy to be advantageous in tying mouse behavior to inter-area network interactions within the brain, and Ryu *et al.* [22] measured the migration of GFP-tagged stem cells in the bladder wall of rats by open intravital microscopy and by transurethral microendoscopy. Beyond the constraints of microendoscopy, Barson *et al.* [23] combined intercranial microendoscopy with a second mesoscale optical axis to investigate cortical architecture, and perhaps most impressively, Xu *et al.* [24] combined high-speed videography with two-photon microendoscopy of calcium signals to connect whisker inputs and dendritic signaling. More recently, de Groot *et al.* [8] demonstrated a dual microscope setup to perform the first concurrent cellular resolution recordings from cerebellum and cerebral cortex in unrestrained mice. In mouse cerebral imaging, the proposed device is well-suited to this sort of experiment. Stereotaxic mounts that may make the present device suitable for dual-site cerebral imaging or ocular imaging have been previously demonstrated [25].

The present research is not without limitations. Most severely, the applicability of the device is limited by the quality of the microendoscope probes, especially the resolution of fiber bundles. As can be seen starkly in Figure 4b, resolution in the red channel was highly constrained (even relative to FOV) due to the pitch of the fiber bundle (3.3 µm). For microendoscopic applications, this limitation may be resolved through the application of magnifying lenses on the probe tip, combined with fiber bundles of larger diameter. However, the use of more complicated microendoscope probes also limits the reach, increases the price, and reduces the number of potential users of the device. Making microendoscope probes for intravital imaging can be a challenge, requiring infrastructure and know-how [26].

The device is also limited to some extent by its inherent geometry. Inclusion of dichroic mirrors introduces a few aberrations, such the observed tilt of the lateral image field, and lens relays consisting of spherical lenses by their nature introduce some coma (not detected) and barrel or pincushion aberration, which we detected when using relay lenses of shorter focal lengths. This may constrain the device to a footprint larger than two equivalent standalone endoscopic probes, although the larger footprint is compensated for by the potential to harness an existing microscope base for truly simultaneous intravital imaging.

The geometry of the dichroic mirror limits direct imaging to fields of view at a 90° angle to each other, and the use of objectives limits direct imaging to locations separated by the length of the objectives, which conventionally have parfocal lengths of at least 45 mm. However, many of these geometric limitations may be overcome when using flexible bundle microendoscope probes, as has been demonstrated by simultaneous imaging of the mouse liver and kidney. The strict minimum limit to the spatial separation between imaging targets when imaging parallel fields of view is thus the width of the microendoscope probe, and flexible probes placed orthogonally may probe the same tissue. Therefore, use of the device faces comparable geometric limitations to the use of multiple standalone endoscope probes. Two-channel fluorescence imaging may even be achieved on the same endoscope probe if the distal dichroic mirror is removed or replaced with a first-surface mirror, provided the fluorophores are suited for simultaneous multi-channel illumination.

While the fiber bundle microendoscope probe was limited by resolution, the GRIN probe was also limited by background fluorescence. Although subcellular features of the liver appear clearly in Figure 5a, these detailed features were not clearly visible prior to background subtraction. Dependence of the system upon image processing is undesirable, as any processing has a risk of adding artifacts, and background subtraction in particular assumes that the size of image’s relevant features is known in advance.

The system as currently applied also struggles to compensate for physiological motion. Despite liver and kidney extravasation, manual gating of images was used to obtain images of high quality. Thus it would be wise to apply the system in tandem with known methods for vibration isolation, such as the grid stabilizer [27] or the tissue window [28], although the selection of stabilization method depends on the tissues of interest [29].

The method of spectral multiplexing may be scaled up to a larger number of channels, and is limited only by illumination intensity and available filters. For additional spectral range, additional non-overlapping fluorophores may be added along with corresponding dichroic mirrors in sequence. Of particular interest is the FDA-approved near-infrared fluorescent contrast agent indocyanine green [30], which would be accessible to a device with two additional filters and two additional dichroic mirrors. Although at present this would require an additional imaging device per additional channel, as a workaround, the system may be extended with filter wheels which enable less simultaneous but lower-cost imaging from multiple locations. Other extensions into temporal modalities and temporal multiplexing are also worth considering: for example, an LED illuminator flickering at high frequency may allow for fluorescence lifetime imaging or flickering of LEDs in the illuminator at different wavelengths may remove the need for separate imaging cameras entirely.

The method outlined in this paper is well-suited to adaptation in many preclinical studies. In particular, potential users may be interested combining multiple narrow-diameter microendoscopic probes with small-footprint tissue immobilization [28,31] for simultaneous longitudinal intravital imaging of multiple stabilized tissues or organs, or with conventional stereotaxic techniques [32] to attain simultaneous multiple views of neural signaling [8]. The system may potentially find application with head mount stereotaxic devices which allow the free movement of mice during microendoscopic imaging [25].

While the present device is limited to preclinical applications, the clinical literature contains several procedures that may benefit from multiplexing of endoscopes. In particular, multiplexing of dual endoscope procedures may reduce their clinical costs and increase their accessibility.

## 5. Conclusions

An endoscopic relay system which attaches to a microscopic base has been presented which supports dual-site fluorescence microendoscopy *in vivo* via spectral multiplexing, allowing simultaneous fluorescent imaging from multiple endoscopic probes. Multiplexing and demultiplexing of fluorescence signals is achieved by dichroic mirrors, and has been demonstrated with Texas Red, FITC, and GFP. The system is compatible with both rigid and flexible microendoscope probes, achieves sub-micron resolutions without probes, and subcellular resolutions through the probes. As fluorescence imaging channels are highly independent, microendoscope probe resolution and imaging capability was not negatively impacted by the reduced spectral range on each imaging channel.

## Data Availability

The data presented in this study are openly available in FigShare at https://doi.org/10.6084/m9.figshare.21671618.v1.
